# Preliminary Study of Real-Time Detection of Chicken Embryo Viability Using Photoplethysmography

**DOI:** 10.3390/s26020472

**Published:** 2026-01-10

**Authors:** Zeyu Liu, Zhuwen Xu, Yin Zhang, Hui Shi, Shengzhao Zhang

**Affiliations:** School of Biomedical Engineering, Anhui Medical University, Hefei 230032, China; 2345012573@stu.ahmu.edu.cn (Z.L.); 2345012539@stu.ahmu.edu.cn (Z.X.); 2345012538@stu.ahmu.edu.cn (Y.Z.)

**Keywords:** chicken embryo viability detection, real-time, continuous multi-day monitoring, photoplethysmography, Short-Time Fourier Transform

## Abstract

Currently, in influenza vaccine production via the chicken embryo splitting method, embryo viability detection is a pivotal quality control step—non-viable embryos are prone to microbial contamination, directly endangering the vaccine batch quality. However, the predominant manual candling method suffers from unstable accuracy and occupational visual health risks. To address this challenge, we developed a novel real-time embryo viability detection system based on photoplethysmography (PPG) technology, comprising a hardware circuit for chicken embryo PPG signal collection and customized software for real-time signal filtering and time–frequency-domain analysis. Based on this system, we conducted three pivotal experiments: (1) impact of the source–detector spatial arrangement on PPG signal acquisition, (2) viable/non-viable embryo discrimination, and (3) embryo PPG signal detection performance for days 10–14. The experimental results show that within the sample size (15 viable, 5 non-viable embryos), the system achieved a 100% discrimination accuracy; meanwhile, it realized 100% successful multi-day (days 10–14) PPG signal capture for the 15 viable embryos, with consistent performance across the developmental stages. This PPG-based system overcomes limitations of traditional and existing automated methods, provides a non-invasive alternative for embryo viability detection, and presents significant implications for standardizing vaccine production quality control and advancing optical biosensing for biological viability detection.

## 1. Introduction

In the influenza vaccine manufacturing industry, the chicken embryo splitting technique has remained the predominant production method since its inception in the 1940s. This technology has achieved global adoption due to its maturity, process stability, and cost-effectiveness. A pivotal quality control step in this production chain involves embryo viability detection, which is implemented at two critical junctures: pre-incubation and post-incubation. This screening process aims to eliminate non-viable embryos, including deceased or compromised ones. These non-viable embryos are susceptible to microbial contamination and exhibit diminished viral replication capacity—two factors that can compromise the quality of the final vaccine batch [1]. Presently, most viability detections continue to depend on manual candling procedures, wherein technicians examine chicken embryos under illuminated conditions in darkened environments, evaluating the embryo viability through visual inspection of the vascular network integrity and clarity. However, the reliability of this manual approach is inherently constrained by operator expertise and the ambient working conditions, resulting in inconsistent detection accuracy and suboptimal processing rates [2,3]. Furthermore, extended periods of candling operations in low-light environments pose substantial occupational hazards to visual health.

To overcome these limitations, researchers have explored various automated approaches for embryo viability detection. Qi-Lei Xu et al. proposed a machine-vision-based detection method for evaluating embryo viability [4]. Their system incorporated a specialized vascular image acquisition apparatus and implemented a SUSAN-based edge detection algorithm to eliminate white-spot artifacts originating from eggshell pores, thereby achieving high-fidelity embryo vascular imaging [5]. By utilizing vascular morphological features as discriminative inputs, they adopted a nearest-neighbor classifier for viability status determination. Although machine vision has demonstrated remarkable efficacy in applications such as defect inspection and autonomous vehicles, the study failed to account for two critical aspects in embryo evaluation: (1) the temporal delay between embryonic death and observable vascular degeneration, and (2) heterogeneous vascular distribution patterns that may result in detection failures with fixed-angle imaging configurations—both of which could potentially compromise the identification accuracy [6].

Wang et al. developed an embryo viability prediction method by monitoring respiratory CO_2_ emissions, finding significant differences between viable/non-viable embryos on days 1–5. Using RF and SVM classifiers, they achieved 100% accuracy, but the need for individual sealed chambers and prolonged monitoring limits it to small-scale use, making it incompatible with rapid vaccine production [7].

To address inefficient manual candling, Liu and Ngadi applied near-infrared hyperspectral imaging to distinguish between fertile and non-fertile chicken eggs. By analyzing spectral transmission characteristics (MS/MG) and using PCA–K-means, the system achieved 100% accuracy for pre-incubation (day 0) fertility detection (via MS’s top three bands) and up to 84.1% accuracy for day 4 embryo development detection, demonstrating hyperspectral imaging’s utility for non-destructive egg-hatching property assessment [8,9]. Rahmat et al. (2023) proposed a system for classifying fertile and infertile eggs using thermal camera images: they used ROI to reduce the background interference and histogram analysis to identify RGB color differences between fertile and infertile eggs. Testing on single/group egg images yielded 93.7% accuracy on incubation day 9, demonstrating the method’s potential for efficient egg fertility detection in the poultry industry [10].

Geng and Yu’s teams pioneered embryo viability detection via PPG signals using raw data and convolutional neural networks to classify viable and non-viable embryos with balanced speed and accuracy. However, Geng’s PPG apparatus had limitations: 30% of the 500 measurements were discarded for poor signal quality, and the rest showed ambiguous signals [11,12]. Youssef et al. (2020) developed a non-invasive photoplethysmography (PPG)-based prototype (Egg-PPG) for the real-time and continuous monitoring of avian embryonic heart rates (HRs) during incubation. The system integrates infrared LEDs as the light source and a photodiode with signal amplification and filtering modules, and employs an algorithm combining continuous wavelet transform (CWT) and normalized spectral entropy for automatic embryonic cardiac wave (ECW) detection and HR calculation. Experimental results with broiler chicken embryos showed the system could reliably detect the ECW during embryonic days 7 to 19, achieving 98.7% accuracy in HR estimation compared with manual counting. This low-cost, non-invasive solution is applicable for experimental research (e.g., developmental biology, cardiovascular studies) and industrial incubation scenarios [13].

Building upon these foundational studies, we developed a high-performance embryo photoplethysmography (PPG) acquisition system that enables the rapid, stable, and precise acquisition of PPG signals exhibiting well-defined waveforms and consistent signal quality. To satisfy the efficiency demands of vaccine manufacturing, the system integrates advanced anti-interference technology to facilitate simultaneous multi-channel measurements. Using this platform, we systematically investigated three critical aspects: (i) optimization of the source–detector positioning, (ii) viability discrimination between viable and non-viable embryos, and (iii) continuous multi-day PPG monitoring. This work provides important methodological references for subsequent research in this field.

## 2. Materials and Methods

### 2.1. Chicken Embryo Preparation

The experimental fertilized chicken embryos were 1–2-day-old indigenous embryos procured from Yin’s Breeding Co., Ltd., Hengshui City, Hebei Province, China. Incubation was performed using constant-temperature water-bath incubators (Hongde Livestock Equipment Co., Dezhou, Shandong, China). A total of 60 fertilized chicken embryos aged 0–2 days were purchased in three sequential batches (20 embryos per batch). After each batch of chicken embryos was sterilized and incubated under standard conditions until day 10, 15 viable embryos (confirmed by manual candling) and 5 non-viable embryos (killed by cryopreservation) were retained. Distinct roles were assigned to each batch based on the system optimization process:

Batch 1 (Debugging Batch 1):

This batch was dedicated to optimizing the system’s light source power. Initial measurements revealed unstable signal acquisition, and after iterative adjustment of the red LED power (driven by the VCCS circuit), stable detection of weak embryonic PPG signals was achieved. The optimized power parameters are as follows:

- The maximum power of the used red LED is 3 W, with a maximum allowable current of 700 mA.
- The VCCS circuit applies a sinusoidal alternating current to the red LED, featuring a peak-to-peak value of 400 mA (biased at 200 mA) and an effective value of approximately 245 mA.

Batch 2 (Debugging Batch 2):

Building on the stable power parameters confirmed in Batch 1, this batch focused on optimizing the spatial arrangement of the light source (red LED) and detector. Comparative measurements of three designed arrangements (a/b/c) were conducted on 15 viable chick embryos. The preliminary results indicated that arrangement (c) yielded the highest signal amplitude and lowest noise, providing the geometric parameter basis for formal experiments. The specific parameters of arrangement (c) are as follows:

- The red LED is placed at the small end of the chick embryo, enabling light to penetrate the embryo along the direction connecting its large and small ends.
- The photodetector is positioned at the waist of the chick embryo, with the vertical extension line of its detection surface forming a 90-degree angle with the light incident direction.

Batch 3 (Formal Experimental Batch):

This batch strictly adopted the optimized system parameters (light source power settings from Batch 1, source–detector spatial arrangement (c) from Batch 2). All key experiments (source–detector arrangement validation, viable/non-viable discrimination, multi-day monitoring) were conducted based on this batch to ensure data reliability.

### 2.2. Detection Principle

The system design employs photoplethysmography (PPG), an established technique for human PPG signal measurement. As light traverses biological tissue, it experiences characteristic attenuation. When illuminating human fingers with specific-wavelength light, attenuation by static tissues (e.g., skin, muscle, and bone) remains constant. In contrast, cardiac-rhythm-induced periodic blood flow variations in vessels produce corresponding light attenuation fluctuations that are detectable as intensity changes at the photodetector. Subsequent optoelectronic conversion yields a human PPG signal [14,15,16]. Analogously, in 10-day-old chicken embryos, static components (eggshell and amniotic fluid) maintain constant light attenuation, whereas vascular pulsations generate detectable optical signal variations, permitting embryonic PPG signal acquisition. This fundamental PPG principle—wherein vascular pulsations induce detectable optical signal variations while static components cause constant light attenuation—underlies our embryonic PPG signal measurement system design.

### 2.3. Hardware Circuitry and Embedded Controller Program Design

**Figure 1:**
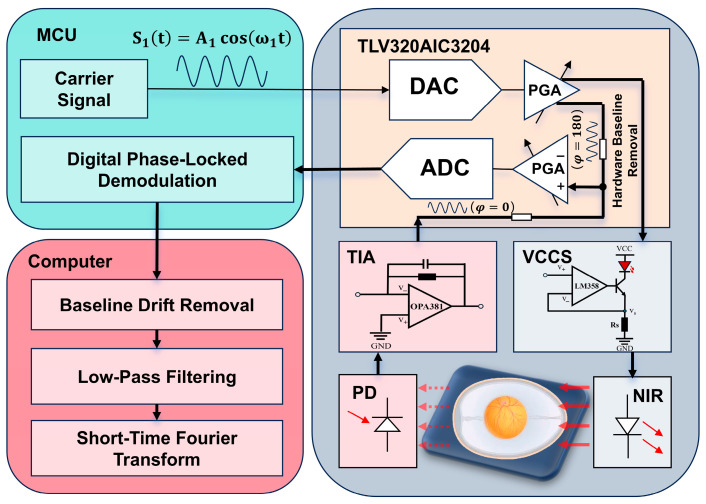
Schematic diagram of the design principle of the chicken embryo viability detection system, showing the core modules (MCU, AD/DA conversion circuit, etc.) and signal-processing method.

**Figure 2:**
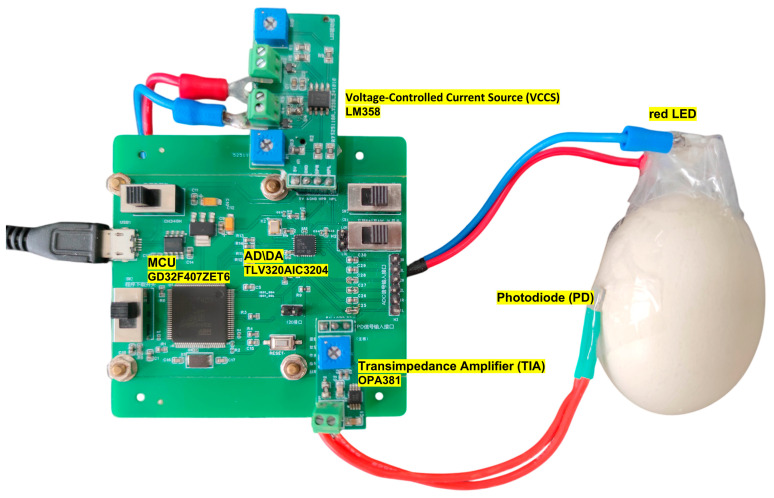
Acquisition and processing circuit for chicken embryo PPG signals.

To ensure the stable and high-fidelity acquisition of weak embryonic photoplethysmography (PPG) signals, this study adopts a modulation–demodulation strategy based on a digitally implemented phase-sensitive detection (PSD) algorithm. Figure 1 illustrates the overall hardware architecture and signal flow, while Figure 2 shows the photograph of the implemented experimental hardware setup.

#### 2.3.1. Modulated Optical Excitation and Carrier Signal Generation

The system employs a microcontroller unit (MCU, GD32F407ZET6, GigaDevice, Beijing, China) to generate a sinusoidal carrier signal used for optical modulation. The carrier frequency was set to 2 kHz, and the peak-to-peak output amplitude was 1.8 V. The carrier frequency of 2 kHz was chosen to be sufficiently higher than the low-frequency ambient light fluctuations (≤200 Hz), while remaining well within the modulation bandwidth of the LED–photodiode pair and the analog front-end, thereby avoiding excessive phase distortion and signal attenuation.

The digital carrier waveform is generated internally by the MCU using a lookup table and is output through the DAC interface of the TLV320AIC3204 audio codec (Texas Instruments, Dallas, TX, USA) via the I^2^S communication protocol. The DAC output is subsequently delivered through the HPL/HPR pins of the codec, producing a continuous 2 kHz sinusoidal voltage waveform.

This sinusoidal voltage signal is applied to the input of a voltage-controlled current source (VCCS) constructed using an LM358 operational amplifier and a TIP41 power transistor. The VCCS converts the voltage modulation into a proportional sinusoidal current, which drives a red LED (wavelength 670–680 nm). As a result, the emitted optical intensity varies sinusoidally at the carrier frequency, forming a modulated light source. Such modulation shifts the PPG signal away from low-frequency ambient light interference and improves the detection robustness [17].

#### 2.3.2. Photodetection and Analog Front-End Signal Conditioning

After transmission through the chicken embryo, the modulated light is received by a silicon photodiode (PD). Due to the photoconductive effect, the PD converts the incident modulated optical signal into a corresponding current signal containing the embryonic PPG information.

To facilitate digital processing, the weak photodiode current is converted into a voltage signal using a transimpedance amplifier (TIA) based on the OPA381, which is specifically designed for low-noise, high-bandwidth current-to-voltage conversion. The resulting voltage signal preserves the carrier modulation while embedding the physiological PPG amplitude variations.

The conditioned analog signal is fed into the IN1_L input of the TLV320AIC3204 and digitized by its integrated ADC. The ADC sampling rate was configured to 96 kHz, providing sufficient oversampling relative to the 2 kHz carrier to ensure accurate digital demodulation and minimize quantization and phase errors.

#### 2.3.3. Embedded Digital Demodulation Based on Phase-Sensitive Detection

Following analog-to-digital conversion, the digitized modulated PPG signal X[n] ($X\left[n\right]=A\times \sin\left(2\pi \times \frac{f}{fs}\times n+\varphi \right)+noise$) is transmitted to the MCU via I^2^S and stored in a pre-allocated buffer. Here, A represents the amplitude of the PPG signal, f is the carrier signal frequency, fs is the sampling rate, n is the index of the current sample point, φ denotes the phase offset, and noise represents additive noise. The MCU then performs digital demodulation using a phase-sensitive detection method equivalent to a digital lock-in amplifier.

Specifically, the MCU internally generates two orthogonal reference signals synchronized with the carrier frequency: a sine reference S[n] ($S\left[n\right]=\sin\left(2\pi \times \frac{f}{fs}\times n\right)$) and a cosine reference C[n] ($C\left[n\right]=\cos\left(2\pi \times \frac{f}{fs}\times n\right)$). These reference signals are precomputed and stored in memory. In the above expressions, f is the reference signal frequency, which is equal to the carrier signal frequency; fs is the sampling rate; and n is the index of the current sample point.

The demodulation process consists of multiplying the acquired signal X[n] with each reference signal and accumulating the results over a fixed window length. Mathematically, this operation performs frequency translation and correlation projection, while the accumulation acts as a narrowband low-pass filter centered at the carrier frequency. This process yields the in-phase (I) ($I=\sum_{n=0}^{N-1}X[n]\cdot S[n]$) and quadrature (Q) ($Q=\sum_{n=0}^{N-1}X[n]\cdot C[n]$) components of the modulated PPG signal. Here, N denotes the total number of samples accumulated within one demodulation window, and n represents the discrete-time index of the current sample.

The PPG amplitude is then computed using

(1) $$A=\sqrt{I^{2}+Q^{2}}$$

This amplitude represents the envelope of the embryonic PPG signal and is inherently robust against ambient light fluctuations, power-line interference, and optical crosstalk between channels.

#### 2.3.4. Data Transmission and System Workflow

After demodulation, the extracted PPG amplitude values are transmitted from the MCU to a host computer via a serial communication interface for further visualization and time–frequency analysis. The complete system workflow—involving optical modulation, photodetection, digital demodulation, and data transmission in turn—is fully implemented on the embedded platform, enabling real-time and continuous PPG monitoring of chicken embryos.

### 2.4. Signal Filtering and Time–Frequency-Domain Analysis

The time–frequency characteristics of embryonic PPG signals constitute essential biomarkers for viability evaluation. However, physiologically induced baseline drift, together with inevitable high-frequency noise during signal acquisition, can substantially compromise time–frequency analysis [18,19]. To mitigate these issues, signal processing was implemented in MATLAB(R2024a) as follows:

(1) A zero-mean triangular window was employed for cross-correlation-based baseline drift suppression [20]. The window length was empirically selected as 10 samples to effectively remove slow-varying baseline components while preserving the pulsatile PPG waveform. Preliminary tests with different window lengths (e.g., 6, 10, 24, and 30 samples) indicated that a length of 10 samples achieved effective baseline suppression without introducing distortion to the pulsatile component, and was therefore adopted in this study.

(2) A 100th-order low-pass FIR filter (cutoff frequency = 7 Hz), designed using the fir1 function, was applied to attenuate high-frequency artifacts. This filter was employed instead of an IIR filter primarily to preserve the linear-phase property of the PPG signals [21]. Since subsequent analysis relies on the Short-Time Fourier Transform (STFT) to characterize time–frequency features, maintaining phase linearity is critical to avoid waveform distortion and frequency smearing. The filter order was empirically chosen to provide sufficient stopband attenuation for high-frequency artifacts while maintaining a narrow transition band around the 7 Hz cutoff. Given the low-frequency nature of embryonic PPG signals and the offline MATLAB(R2024a)-based processing framework, the higher filter order does not impose practical computational constraints.

**Figure 3:**
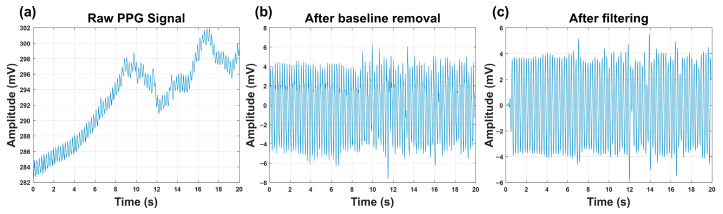
Effects of baseline drift removal and filtering on the PPG signal. (**a**) Raw PPG signal containing low-frequency baseline drift. (**b**) Signal after baseline drift removal using cross-correlation with a zero-mean triangular waveform. (**c**) Signal after subsequent bandpass filtering.

The effectiveness of baseline drift removal and filtering is visually illustrated in Figure 3 by comparing the raw and processed PPG signals.

(3) Subsequently, Short-Time Fourier Transform (STFT) analysis was performed using the tfrstft function with a 255-point Hanning window to generate the time–frequency distribution.

### 2.5. Study on Source–Detector Arrangement for Embryonic PPG Signal Measurement

**Figure 4:**
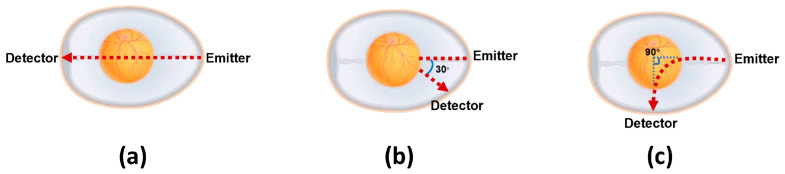
Light source–detector spatial arrangements design. (**a**) The extended vertical lines of the emitter and the detector form a 180-degree angle. (**b**) The extended vertical lines of the emitter and the detector form a 30-degree angle. (**c**) The extended vertical lines of the emitter and the detector form a 90-degree angle.

The spatial geometry of the source–detector pair substantially determines the embryonic PPG signal fidelity, necessitating comprehensive optimization of the optoelectronic arrangement (light source–photodiode spatial relationship). As shown in Figure 4, building upon validated methodologies and incorporating embryonic morphological characteristics, we developed three specialized source–detector arrangements for embryonic detection.

In this study, PPG signals were acquired from identical embryos using three distinct source–detector arrangements, with subsequent comparative analysis to identify the relatively outstanding geometric arrangement for embryonic PPG signal detection within our experimental system.

### 2.6. Comparative Analysis of PPG Signals Between Viable and Non-Viable Chicken Embryos

The primary objective of this study is to enable rapid viability detection of embryos during vaccine production, permitting the timely elimination of non-viable embryos. After determining the relatively outstanding source–detector arrangement for embryonic PPG signal acquisition, we performed comparative PPG measurements between viable and non-viable embryos. To ensure analytical rigor and industrial applicability, we conducted a time–frequency analysis of embryonic PPG signals using Short-Time Fourier Transform (STFT) in MATLAB(R2024a). This method enables more precise and efficient discrimination between viable and non-viable embryos from a frequency-domain perspective.

### 2.7. Continuous Multi-Day Monitoring of Embryonic PPG Signals

In influenza vaccine production, 9–10-day-old embryos are routinely pre-incubated for 24 h prior to the candling inspection and viral inoculation of qualified embryos. Following inoculation, a secondary (strain-dependent) incubation period of 48–72 h is implemented, after which a second candling inspection precedes viral fluid harvesting from viable embryos. This standardized protocol requires accurate viability detection of embryos aged 10–14 days.

To address this industrial need, we performed a 5-day continuous monitoring study to identify potential challenges in embryonic PPG signal acquisition during prolonged incubation. All experimental embryos were individually labeled and measured daily under rigorously controlled conditions, including fixed time intervals, consistent measurement sites, standardized detection angles, and uniform illumination intensity. This experimental design aimed to minimize confounding variables that could compromise the signal quality.

## 3. Results and Discussion

### 3.1. Analysis of Source–Detector Arrangements

**Figure 5:**
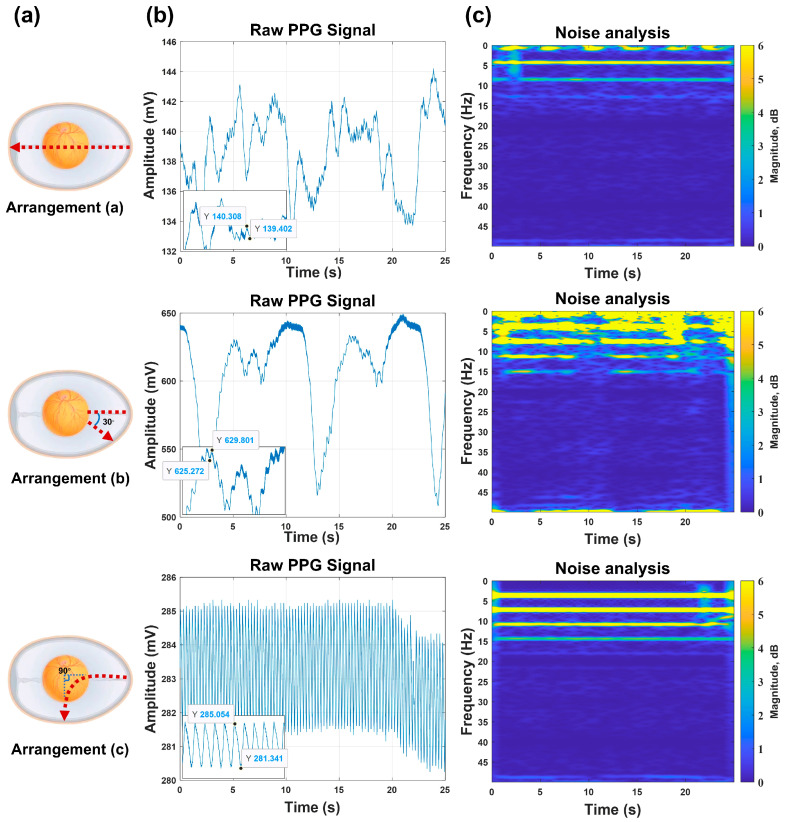
Comparison of measurement results for different light source–detector arrangements. Column (**a**): Different light source–detector arrangements. Column (**b**): Original chicken embryo PPG signals data under different light source–detector arrangements, with a locally enlarged view of the signal shown at the bottom-left corner of each subfigure. Column (**c**): Analysis of the measurement noise for different light source–detector arrangements.

This experiment was conducted on 15 viable embryos from Batch 3, aiming to validate the relatively outstanding source–detector arrangement. PPG signals were acquired under three arrangements while maintaining consistent system parameters (power, detection angle, etc.). Figure 5 shows that the detection system successfully captured embryonic PPG signals across all arrangements, with clearly observable inter-arrangement variations. These differences were primarily reflected in the PPG signal amplitude and accompanying noise levels. Through localized waveform magnification and Short-Time Fourier Transform (STFT) analysis on the MATLAB(R2024a) platform, we systematically examined the time–frequency characteristics, enabling a detailed comparison of arrangement-dependent measurement outcomes. Regarding the signal amplitude characteristics, arrangement (a) generated signals with mean amplitudes of approximately 1 mV, while arrangements (b) and (c) exhibited higher signal amplitudes, with peak-to-peak values of approximately 4.5 mV for arrangement (b) and 3.7 mV for arrangement (c). These findings demonstrate that arrangements (b) and (c) provide enhanced detectability for embryonic PPG signals under identical experimental conditions. The superior amplitude renders these arrangements more robust against noise interference. Consequently, based on the amplitude analysis, (b) and (c) constitute superior source–detector arrangements.

Subsequently, we analyzed the noise characteristics of arrangements (b) and (c). The embryonic PPG signals predominantly clustered at approximately 4 Hz, with extraneous frequency components classified as noise. For arrangement (b), (i) substantial noise contamination was observed across the low- and high-frequency bands, (ii) noise components were distributed throughout the 0–15 Hz spectrum, and (iii) noise interference persisted continuously during measurements. In comparison, arrangement (c) demonstrated (i) minimal low-frequency noise contamination and (ii) well-defined high-frequency noise components that were readily eliminable via digital filtering.

A comprehensive evaluation of the signal amplitude and noise characteristics revealed that arrangement (c) exhibited superior performance compared with the other two source–detector arrangements. Therefore, arrangement (c) was selected for all subsequent experimental measurements.

Furthermore, this study systematically investigated the underlying causes of performance variations between the three source–detector arrangements. We hypothesized that these differences may be attributed to three key factors: (1) optical path length, (2) light penetration depth within the embryo, and (3) emitter–receiver separation distance. Arrangement (a) features the longest optical path length, vertically penetrating the entire embryo with the maximal light penetration depth and greatest emitter–receiver separation. This arrangement yields the lowest signal amplitude but demonstrates minimal noise interference. In contrast, arrangement (b) operates in reflection mode with a reduced optical path length, shallower light penetration, and minimal emitter–receiver separation, resulting in the highest amplitude signals but with significant noise contamination. Arrangement (c) exhibits an optical path length comparable with (b), with a light penetration depth intermediate between (a) and (b), and an emitter–receiver separation distance shorter than (a) but longer than (b). This arrangement produces signal amplitudes similar to (b) while maintaining noise levels between those of (a) and (b). Based on these observations, we provide the following qualitative interpretation: (i) an increased optical path length and greater penetration depth may lead to enhanced light attenuation, consequently reducing the signal amplitude, and (ii) a reduced emitter–receiver separation may elevate the measurement noise. Consequently, arrangement (c)’s balanced parameters in all three aspects generate optimal measurements featuring a substantial signal amplitude and suppressed noise levels.

### 3.2. Comparison of PPG Signals Between Viable and Non-Viable Chicken Embryos

**Figure 6:**
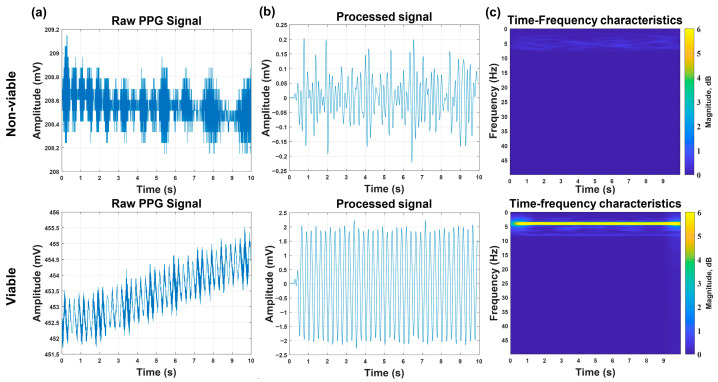
Comparative analysis of PPG signals measurements between viable and non-viable chicken embryos. Column (**a**): Original pulse data of chicken embryos under different active states. Column (**b**): Pulse data of chicken embryos under different active states after filtering. Column (**c**): Analysis of time–frequency characteristics of chicken embryo pulse data in different active states.

Formal discrimination experiments were performed on Batch 3 (15 viable embryos, 5 non-viable embryos) using arrangement (c) and optimized signal-processing algorithms. A 10 s PPG signal was acquired for each embryo, followed by a baseline correction, noise filtering, and STFT analysis. Key findings are shown in Figure 6: For viable embryos, the processed signals exhibited clear periodicity with a characteristic peak at ≈4 Hz in the time–frequency domain [22], and the PPG signal amplitudes exceeded 1 mV; both features were consistent across all 15 viable samples. For non-viable embryos, the raw signals showed irregular high-frequency fluctuations, and the processed signals lacked periodicity with a diffuse energy distribution in the time–frequency domain; the maximum PPG signal amplitude was only 0.4 mV, and all these features were consistent across all five non-viable samples. Based on these features, the system achieved a 100% discrimination accuracy for Batch 3. This high accuracy was attributed to three factors: (1) stable signal acquisition enabled by power optimization (Batch 1); (2) minimal noise interference from arrangement (c) (validated by Batch 3); and (3) distinct signal characteristics between viable embryos (pulsatile) and cryopreserved non-viable embryos (non-pulsatile), reducing the misclassification risks.

### 3.3. Continuous Monitoring of PPG Signals in Viable Embryos

**Figure 7:**
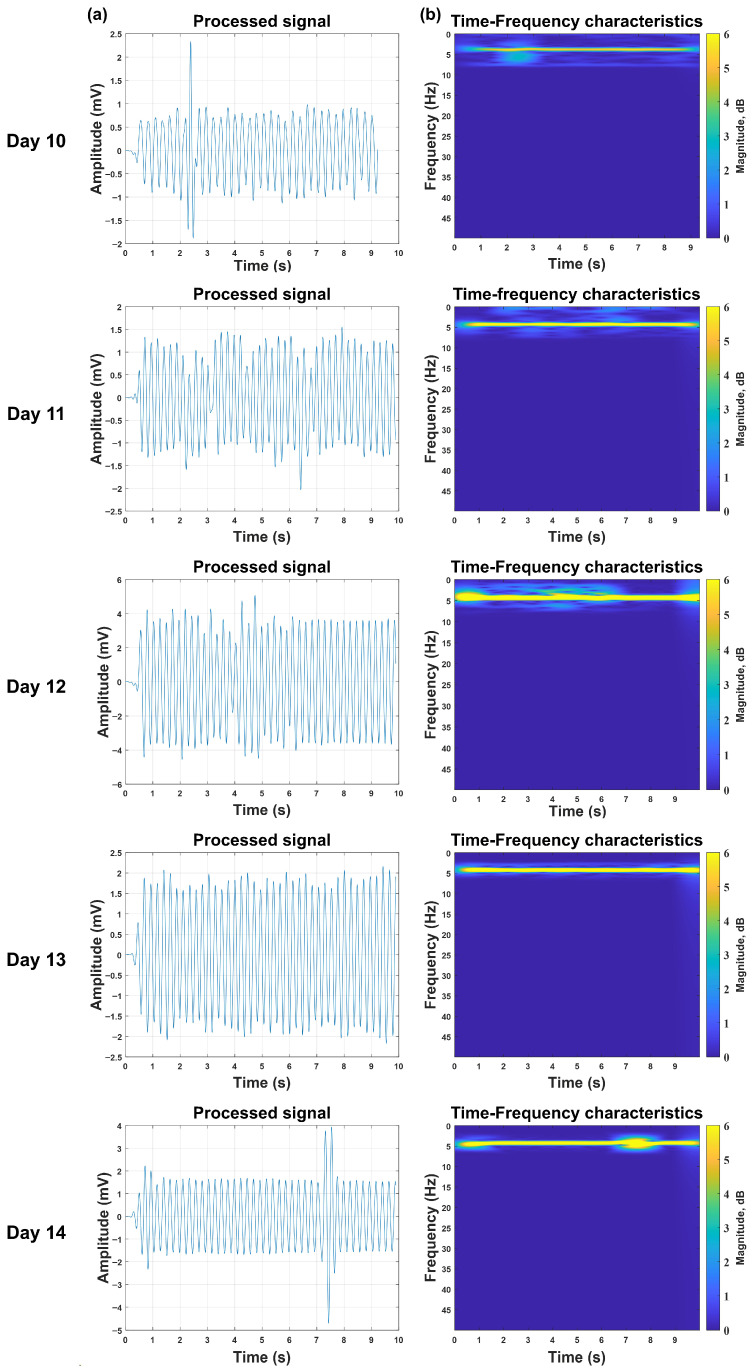
Monitoring the PPG signals of viable chicken embryos for five consecutive days. Column (**a**): Pulse data of viable chicken embryos during incubation days 10 to 14. Column (**b**): Analysis of the time–frequency characteristics of chicken embryo pulse data during incubation days 10 to 14.

Continuous 5-day monitoring (days 10–14) was conducted on the 15 viable embryos from Batch 3, with strictly controlled experimental conditions (fixed daily measurement time, consistent detection site, uniform illumination intensity) to minimize confounding variables. This investigation longitudinally tracked the PPG signals in individual embryos from days 10 to 14 to validate the system’s viability detection capability across the varying incubation periods required for different viral strains (e.g., 48 h for influenza A virus, corresponding to days 10–12 of incubation; 72 h for influenza B virus, corresponding to days 10–14). Notably, the system achieved 100% successful acquisition of pulsatile PPG waveforms for all 15 viable embryos throughout the entire 10–14-day observation window—no signal loss or failure to detect was recorded for any individual embryo on any monitoring day. In this single sample, the initial measurements on day 10 revealed a relatively low signal intensity, potentially reflecting underdeveloped cardiovascular function at this early developmental stage. The signal amplitude progressively increased through days 11–12, peaking at day 12, presumably due to maturation of the embryonic pulsatile activity. Subsequently, days 13–14 exhibited declining signal strength, likely resulting from increased optical attenuation through the enlarging embryonic tissue. The PPG signal amplitudes of the remaining chick embryo samples also showed a similar variation trend, but the incubation days when they reached the amplitude peaks varied, see Figure 7.

## 4. Conclusions

This study addressed the critical bottlenecks of embryo viability detection in influenza vaccine production by developing a high-performance, non-invasive system grounded in photoplethysmography (PPG) technology. By integrating optimized hardware design and advanced signal processing, the proposed system potentially mitigates the inherent limitations of manual candling and existing automated methods, offering a reliable, real-time solution for quality control in biopharmaceutical manufacturing. Rather than proposing a new detection principle, this study focused on improving the signal robustness, long-term stability, and industrial applicability of PPG-based embryo viability detection. The current system represents a laboratory-scale prototype, and further integration and engineering optimization are required for large-scale industrial deployment.

A core innovation of this work lies in the systematic optimization of PPG signal acquisition. Through iterative testing of three source–detector spatial arrangements, we identified arrangement (c) (the light source is located at the small end of the chicken embryo, and the light receiver is positioned on the lateral side, with a 90° angle between their extension lines) as the relatively outstanding configuration, which balances the signal amplitude and noise suppression, thereby alleviating the signal quality issues reported in prior PPG-based studies (e.g., high sample discard rates due to substandard signals). This optimization, combined with digital phase-locked demodulation and a multi-step signal-processing pipeline (baseline correction via zero-mean triangular cross-correlation, 100th-order low-pass FIR filtering, and Short-Time Fourier Transform (STFT) analysis), ensures the high-fidelity capture and characterization of embryonic PPG signals.

Compared with vision-based automated methods that rely on vascular morphology, the proposed PPG-based approach is inherently driven by pulsatile physiological signals rather than static vascular images. In principle, this signal mechanism is less affected by the temporal delay between embryonic death and observable vascular degeneration. In addition, because the method does not depend on capturing a specific vascular imaging plane, it is expected to be less sensitive to heterogeneous vascular distribution patterns within the embryo.

Experimental validation confirms the system’s robust performance across key industrial requirements. In the viability discrimination tests using 15 viable and 5 non-viable embryos, the system achieved 100% accuracy within the tested samples by leveraging distinct signal features: viable embryos exhibited periodic waveforms with a characteristic ≈4 Hz peak in the time–frequency domain and amplitudes exceeding 1 mV, while non-viable embryos showed irregular fluctuations, diffuse energy distribution, and maximal amplitudes of only 0.4 mV. Longitudinal monitoring of 10-to-14-day-old embryos further demonstrated that the chick embryo viability detection capability of this system can cover the entire incubation window for vaccine production.

Despite these strengths, limitations remain. The present study did not explicitly quantify post-mortem temporal delay effects or systematically evaluate different vascular distribution scenarios, and the modest sample size warrants validation with larger cohorts. Addressing these aspects in future work will further clarify the extent to which the proposed approach mitigates the limitations of existing automated detection methods and will solidify its role in bioprocessing and biosensing applications.

## Data Availability

In this paper, the research data are shared via the open database Zendo through the following DOI: https://doi.org/10.5281/zenodo.17563198, or the following website: The data and program of “Preliminary Study of Real-time Detection of Chicken Embryo Viability Using Photoplethysmography”.

## Abbreviations

The following abbreviations are used in this manuscript:

PPG	Photoplethysmography
STFT	Short-Time Fourier Transform
LED	Light Emitting Diode
VCCS	Voltage-controlled current source
MCU	Microcontroller unit
ADC	Analog-to-Digital Converter
DAC	Digital-to-Analog Converter
PGA	Programmable Gain Amplifier
TIA	Transimpedance amplifier
PD	Photodiode
FIR	Finite Impulse Response
